# Moving

**Published:** 1917-04

**Authors:** 


### Moving.

Think carefully before you move to:

A district entirely out of convenient reach of your present practice;

The country from the city, or vice versa, unless you are somewhat familiar with the different conditions or are young enough to adapt yourself to them;

A residence which necessitates the maintenance of a separate office, at a considerable distance, if your practice is largely emergent and, in any case, unless you have thoroughly considered the loss of time and increased expense;

A big house, deserted by an aristocratic family, unless you are sure that you are not buying at the beginning of an exodus of the former inhabitants of the district and the transition period of boarding and rooming houses which usually lasts for many years before business comes in;

A house, all right in itself but off of traveled routes.

A district promising well for business purposes but involving such environment and so late office hours that, however successful you may be, success will be untranslatable into proper enjoyment of life;

A district not sufficiently central for developing a special practice if that is your object;

A location too far down town for a reasonable chance of building up a stable, desirable general practice, if your inclinations and abilities are of this nature;

A neighborhood in which racial, religious and other affiliations are so strong as to constitute a handicap to one not included in these affiliations;

A district well adapted to your desires but in which the population is fixed in its professional allegiance;

A "growing section" which may not grow;

In short, don't drop a reasonably satisfactory certainty for a gamble.

